# Functional Pharmacological Validation of Betahistine Release from Chitosan Microspheres Using an Isolated Guinea-Pig Gastric Smooth-Muscle Model

**DOI:** 10.3390/pharmaceutics18101339

**Published:** 2026-10-21

**Authors:** Bissera Pilicheva, Plamen Zagorchev

**Affiliations:** 1 Department of Pharmaceutical Sciences, Medical University Pleven, 5800 Pleven, Bulgaria; bisera.pilicheva@mu-pleven.bg; 2 Department of Biomedical Sciences, Medical University Pleven, 5800 Pleven, Bulgaria

**Keywords:** betahistine, chitosan microspheres, modified drug release, functional pharmacological assessment, ex vivo model, gastric smooth muscle, drug-release kinetics, pharmacological activity

## Abstract

**Background:** Betahistine-loaded chitosan microspheres have previously demonstrated formulation-dependent drug release; however, conventional in vitro release testing does not establish whether the drug incorporated into the delivery system retains pharmacological activity. **Methods:** This study evaluated the time-dependent functional activity of four previously characterized betahistine-loaded chitosan formulations using isolated guinea-pig gastric smooth muscle strips. Microsphere quantities corresponding to an equivalent nominal amount of 10 mg betahistine were compared with free betahistine. Spontaneous contractile activity was continuously recorded and mean peak contractile force (F*avr*) and the area under the F*avr*–time curve were evaluated. **Results:** Free betahistine produced a pronounced early response followed by progressive attenuation. In contrast, all microsphere formulations produced gradually increasing and sustained responses, with higher observed F*avr* values than free betahistine at later observation times. Time-matched analysis revealed positive formulation-dependent associations between cumulative betahistine release and F*avr* (R^2^ = 0.8384–0.9914), whereas functional performance did not follow the rank order of drug loading. **Conclusions:** These findings provide functional evidence consistent with sustained availability of pharmacologically active betahistine from the chitosan microsphere formulations and support ex vivo functional assessment as a complementary approach to conventional in vitro release characterization.

## 1. Introduction

Betahistine dihydrochloride (BH) is a structural analogue of histamine that has been widely used in the management of vestibular disorders, particularly Ménière’s disease. Betahistine exhibits relatively weak agonistic activity at histamine H1 receptors and more pronounced antagonistic activity at H3 receptors. However, its interactions with histamine receptor subtypes may vary depending on the experimental system [1,2]. The pharmacological properties of betahistine have been investigated using a variety of experimental models, including isolated smooth-muscle preparations, providing evidence that histaminergic mechanisms can be associated with measurable changes in contractile activity [3]. Histamine-responsive smooth-muscle preparations may therefore provide a useful functional model for assessing whether betahistine released from a drug-delivery system retains pharmacological activity.

Modified-release drug-delivery systems are designed to regulate the rate and duration of drug availability and thereby modify the temporal pattern of pharmacological exposure. Polymeric microspheres are attractive for this purpose because formulation variables such as polymer composition, polymer concentration and drug-to-polymer ratio can influence drug incorporation, matrix structure and subsequent drug release. Chitosan is particularly suitable for pharmaceutical applications because of its biocompatibility, biodegradability, and ability to form particulate delivery systems. Depending on their composition and preparation method, chitosan microspheres can provide prolonged drug release, while changes in polymer concentration and drug-to-polymer ratio may substantially modify their physicochemical and release characteristics [4,5,6,7]. Thus, formulation composition may determine both the amount of drug incorporated into the microspheres and its subsequent temporal availability.

The biological relevance of the guinea-pig gastric smooth-muscle preparation as a model for histaminergic pharmacology is well established. Histamine produces contractile responses in different regions of the guinea-pig stomach, including the fundus, antrum and pylorus, and these responses have been shown to be abolished by the H1-receptor antagonist mepyramine, demonstrating the involvement of H1 receptors in the contractile response [8]. Studies performed using isolated guinea-pig gastric smooth-muscle cells have similarly demonstrated that histamine induces concentration-dependent contraction and increases in intracellular calcium. The H1 agonist pyridylethylamine produces a comparable response and the H1 antagonist pyrilamine inhibits the response to histamine [9]. Further experimental evidence has demonstrated that both the contractile response and histamine-stimulated inositol phosphate formation in guinea-pig gastric smooth muscle are mediated predominantly through H1 receptors [10]. These findings provide a pharmacological basis for using isolated guinea-pig gastric smooth muscle as a functional model in which histaminergic activity can be monitored through changes in contractile force.

In a previous study, we developed and characterized betahistine-loaded chitosan microspheres prepared by a W/O emulsion solvent-evaporation technique [11]. Four formulations, designated M1–M4, were obtained by varying the amounts and ratios of betahistine and chitosan. The formulations differed in their physicochemical characteristics, including mean particle size, drug loading and entrapment efficiency, and exhibited formulation-dependent release profiles. Mean particle size ranged from 3.82 to 7.69 μm, while entrapment efficiency ranged from 69.37% for M1 to 98.27% for M4. The microspheres provided prolonged betahistine release over the 10 h experimental period, with release characteristics dependent on formulation composition [11]. Importantly, the previous release experiments were performed using quantities of microspheres corresponding to an equivalent nominal amount of 10 mg betahistine, providing a basis for comparison with the functional experiments performed in the present study.

Although in vitro release testing is an essential component of the characterization of modified-release particulate systems, it primarily describes the transfer of drug from the delivery system into the release medium. Such testing provides information on the kinetics and extent of drug liberation but does not establish whether the released drug retains its pharmacological activity or whether differences in release behavior are reflected in differences in biological response. Current approaches to the evaluation of biopolymeric particulate systems therefore emphasize the value of release methods with greater biological relevance and of considering drug release as one component of overall delivery-system performance [12]. This distinction is particularly relevant to drug-loaded polymeric microspheres, in which drug liberation from the matrix and subsequent interaction of the released drug with a biological target represent sequential but distinct processes.

Functional pharmacological assays can therefore provide complementary information to conventional in vitro release testing. By monitoring a biological response following exposure to a drug-delivery system, such assays can determine whether the active pharmaceutical ingredient released from the carrier remains capable of producing the expected pharmacological effect. Previous studies of biodegradable microspheres have shown in other drug systems that prolonged in vitro drug release may be associated with sustained pharmacokinetic exposure and prolonged pharmacological responses in vivo [13,14,15]. These studies demonstrate the value of complementing drug-release characterization with biological assessment. However, the relationship between in vitro release and biological activity depends on the drug and formulation and cannot be inferred from the release profile alone. Against this background, functional assessment of betahistine-loaded chitosan microspheres using a histamine-responsive smooth-muscle preparation provides an opportunity to determine whether the drug incorporated into the microsphere formulations retains measurable pharmacological activity.

In the present study, the contractile activity of isolated guinea-pig gastric smooth muscle preparations was continuously monitored following exposure to four previously characterized betahistine-loaded microsphere formulations. The formulations were compared using quantities corresponding to the same nominal amount of betahistine, thereby controlling for differences in nominal drug amount while allowing formulation-dependent temporal response profiles to be compared.

The novelty of the present study lies in the time-resolved ex vivo functional assessment of betahistine-loaded chitosan microspheres previously developed and characterized by our group. Whereas the previous investigation focused on formulation development, physicochemical properties, drug loading and in vitro release kinetics, the current work examines the contractile responses of isolated guinea-pig gastric smooth muscle exposed to these formulations. Specifically, we compared the magnitude and temporal characteristics of the responses to free betahistine and four microsphere formulations administered at equivalent nominal drug doses. We also explored formulation-specific temporal associations between previously determined cumulative drug release and the observed functional responses. Thus, the present study extends the physicochemical characterization of these delivery systems by providing complementary evidence of their ex vivo functional activity, without assuming a direct causal relationship between drug release and contractile response.

## 2. Materials and Methods

### 2.1. Betahistine-Loaded Chitosan Microspheres

Betahistine-loaded chitosan microspheres were prepared using an emulsion solvent-evaporation technique, as previously described [11]. Four formulations, designated M1–M4, were selected for the present functional pharmacological study. The initial betahistine-to-chitosan ratios were 1:1 for M1, 1:1.5 for M2, 1:2 for M3 and 1:1 for M4. The corresponding initial quantities of betahistine and chitosan were 100 and 100 mg, 100 and 150 mg, 100 and 200 mg, and 200 and 200 mg, respectively. The experimentally determined drug-loading values were 47.4%, 46.8%, 36.8% and 54.0% for M1, M2, M3 and M4, respectively. These values were used to calculate the mass of each microsphere formulation corresponding to an equivalent nominal amount of 10 mg betahistine. The required mass of microspheres was calculated according to:

mMS = mBH × 100/DL

where mMS is the mass of microspheres used in the experiment, mBH is the nominal amount of betahistine (10 mg), and DL is the experimentally determined drug-loading value (%). Accordingly, 21.10 mg of M1, 21.37 mg of M2, 27.17 mg of M3 and 18.52 mg of M4 were used to provide a nominal betahistine amount of 10 mg for each formulation. The formulations were therefore compared at an equivalent nominal drug dose, whereas the amount of polymeric carrier introduced into the organ bath varied according to the drug loading of each formulation. The free-betahistine group served as the reference group for all functional comparisons. No individual microsphere formulation was used as a reference. Unloaded chitosan microspheres were not included as a separate control group. The physicochemical characteristics and in vitro betahistine-release profiles of these microspheres were determined previously and have been reported elsewhere [11].

### 2.2. Experimental Animals and Preparation of Gastric Smooth-Muscle Strips

The pharmacological activity of betahistine-loaded chitosan microspheres was evaluated using isolated gastric smooth-muscle preparations obtained from male guinea pigs weighing 350 ± 50 g. The animals were maintained under standard laboratory conditions. All procedures involving animals were performed in accordance with the Bulgarian Food Safety Agency guidelines and were approved by the Ethics Committee of the Medical University of Plovdiv, Bulgaria (approval No: 1/22.01.2015). Following laparotomy, smooth-muscle preparations were excised from the middle part of the gastric corpus, in a circumferential orientation relative to the greater curvature. The mucosa was carefully removed, and strips measuring 13.0 ± 1.5 mm in length and 1.5–2.0 mm in width were prepared for the organ-bath experiments. During preparation of the smooth-muscle strips, a physiological solution containing the following components (mmol/L) was used: Na^+^, 143; K^+^, 5.84; Ca^2+^, 3.7; and Cl^−^, 157.

### 2.3. Organ-Bath Conditions

Following preparation, the smooth-muscle strips were transferred individually to organ baths containing modified Krebs solution with the following composition (mmol/L): Na^+^, 143; K^+^, 5.84; Ca^2+^, 2.5; Mg^2+^, 1.19; Cl^−^, 133; HCO_3_^−^, 16.7; H_2_PO_4_^−^, 1.2; and glucose, 11.5. The Krebs solution was continuously aerated with a gas mixture containing 95% O_2_ and 5% CO_2_. The pH was maintained within the physiological range at 7.28 ± 0.08, and the temperature was maintained at 35.5 ± 0.3 °C. One end of each smooth-muscle strip was attached to a fixed glass holder positioned at the bottom of the organ bath, while the other end was connected to a TRI 201 isometric force transducer (LSI LETICA; Panlab S.L., Barcelona, Spain).

### 2.4. Recording System and Measurement of Spontaneous Contractile Activity

Spontaneous contractile activity (SCA) of the isolated smooth-muscle strips was recorded isometrically using a tensometric recording system incorporating a bridge instrumentation amplifier connected to the force transducer. The amplified signal was digitized and continuously recorded using dedicated data-acquisition software. The software enabled real-time visualization of the force signal and subsequent determination of parameters describing spontaneous smooth-muscle contractile activity.

### 2.5. Preparation and Experimental Allocation

A total of 60 isolated gastric smooth-muscle preparations were obtained from six guinea pigs and allocated to five experimental groups: free betahistine (BH) and four betahistine-loaded chitosan microsphere formulations (M1–M4), with 12 preparations per group. Preparations from each of the six animals were included in all five treatment groups. Therefore, each treatment group was represented by six independent animals, although multiple preparations from the same animal were included. The preparations were randomly allocated to the experimental conditions. However, individual animal identifiers were not retained in the final dataset, and the exact number of preparations contributed by each animal to each group could not be reconstructed. Solutions required for the experiments were freshly prepared on the day of use and stored at 5 °C until required. Before administration, the solutions were brought to the appropriate experimental temperature.

### 2.6. Equilibration and Standardization of Mechanical Load

Following an initial preparatory period, during which the solution in each organ bath was replaced twice, each smooth-muscle preparation was subjected to a standardized mechanical load of 7 mN. Preparations were allowed to equilibrate for 30–60 min, until stable spontaneous contractile activity was established.

### 2.7. Verification of Contractile Responsiveness

At the beginning and at the end of each experiment, acetylcholine (ACh; 10 μM; Sigma, Steinheim, Germany) was added to the organ bath as a functional test of tissue responsiveness. Following the initial ACh challenge, the preparations were washed with fresh Krebs solution and allowed to re-establish stable spontaneous contractile activity before baseline recording and administration of the test formulation. Experiments showing a difference greater than 10% in the contractile response to ACh between the initial and final assessments were excluded from the analysis. The ACh response was used as a functional check of the viability and contractile capacity of the preparation before and after the experimental exposure.

### 2.8. Baseline Recording and Quantification of Spontaneous Contractile Activity

Before administration of the test substance or formulation, spontaneous contractile activity was recorded continuously for 5 min to establish the baseline activity of each preparation. For time-course analysis, the force average (F*avr*) was calculated as the arithmetic mean of the peak forces of spontaneous contractions occurring within each predefined 1 min observation interval. F*avr* was used as the primary parameter for characterizing the time-dependent functional response. For each experimental group, the maximum observed value of F*avr* (F*avr*,max) was identified from the investigated time points. F*avr*,max was defined as the highest observed value of mean contractile force (F*avr*) during the 180 min experimental period. This operational definition does not imply that the absolute maximum pharmacological response was reached. The time corresponding to F*avr*,max was recorded as the time of the maximum observed response. When F*avr* continued to increase at the final observation point (180 min), the true time to maximum response was considered not to have been reached within the experimental observation period. The overall magnitude and persistence of the response were additionally characterized by calculating the area under the F*avr*–time curve from 2 to 180 min (AUC_2–180_), expressed as mN·min. Post-treatment responses were evaluated in relation to the corresponding baseline activity recorded for each preparation.

### 2.9. Free Betahistine and Microsphere Formulations

Two types of betahistine exposure were evaluated: free betahistine in solution and betahistine incorporated into chitosan microspheres. For the free-betahistine group, a betahistine solution was added to the organ bath to obtain a final concentration corresponding to 10 mg betahistine in 20 mL of organ-bath medium, i.e., 0.5 mg/mL (500 µg/mL). For the microsphere groups, a predetermined mass of each formulation (M1–M4), corresponding to 10 mg betahistine based on the experimentally determined drug-loading value, was introduced into the organ bath. Thus, the free-betahistine group and all four microsphere groups were compared at an equivalent nominal amount of betahistine. In contrast, the total mass of microspheres differed among M1–M4 because of differences in drug loading. Following administration, the spontaneous contractile activity of the smooth-muscle preparations was continuously recorded.

### 2.10. Comparison with Previously Determined Drug-Release Profiles

The functional response profiles obtained in the present study were compared with the previously reported in vitro betahistine-release profiles of the corresponding microsphere formulations [11]. For each formulation, F*avr* values were paired with the previously determined cumulative betahistine-release values at the four common observation times (20, 60, 120 and 180 min). Because the release and functional experiments were performed independently, these time-matched comparisons were used to examine formulation-specific release–response associations and were not considered a formal in vitro–ex vivo correlation.

### 2.11. Statistical Analysis

Statistical analysis was performed using Statistica 7, Microsoft Excel 7.0 and GraphPad Prism 6.01 (GraphPad Software, Boston, MA, USA). Data are expressed as mean ± standard deviation (SD). Normality of data distribution was assessed using the Kolmogorov–Smirnov test. Differences among experimental groups at the corresponding observation time points were analyzed using one-way analysis of variance (one-way ANOVA), followed by Bonferroni’s post hoc multiple-comparison test. Time-dependent differences within each experimental group were analyzed using repeated-measures ANOVA followed by Bonferroni’s post hoc multiple-comparison test, with the response at 2 min used as the reference.

For the time-matched release–response analysis, linear regression was performed separately for each microsphere formulation using cumulative betahistine release as the independent variable and F*avr* as the dependent variable. The goodness of fit was expressed as the coefficient of determination (R^2^). Because only four common observation times were available for each formulation, the regression analysis was considered descriptive. Statistical significance for the ANOVA-based comparisons was accepted at *p* < 0.05. Statistically significant differences are indicated in the corresponding figures. The individual smooth-muscle preparation was treated as the observational unit (*n* = 12 per treatment group), whereas six animals served as the independent biological sources. Repeated measurements from the same preparation were considered in the time-course analysis. However, because the individual animal of origin was not retained for each preparation, potential within-animal correlations could not be explicitly incorporated into the statistical models. Consequently, the reported *p*-values reflect preparation-level comparisons without adjustment for the clustering of preparations within animals and should be interpreted with caution.

## 3. Results

### 3.1. Composition and Drug-Loading Characteristics of the Betahistine-Loaded Chitosan Microspheres

The four investigated microsphere formulations differed in drug loading, with values of 47.4%, 46.8%, 36.8% and 54.0% for M1, M2, M3 and M4, respectively. M4 therefore exhibited the highest drug loading, whereas M3 showed the lowest. To provide an equivalent nominal amount of 10 mg betahistine in the functional experiments, 21.10, 21.37, 27.17 and 18.52 mg of M1, M2, M3 and M4 microspheres, respectively, were introduced into the organ bath. The formulation characteristics relevant to the present study are summarized in Table 1.

### 3.2. Baseline Spontaneous Contractile Activity of Isolated Gastric Smooth-Muscle Preparations

**Figure 1:**
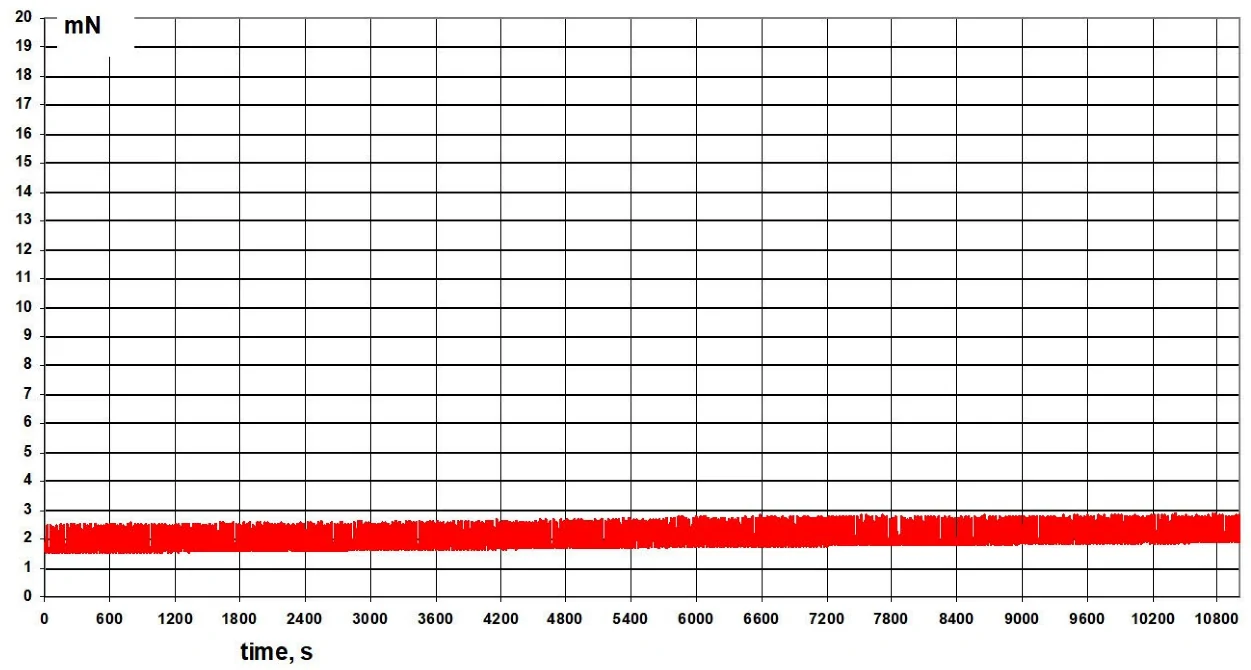
Representative isometric force–time recording of SCA of an isolated guinea-pig gastric corpus smooth-muscle preparation under baseline organ-bath conditions.

Isolated smooth-muscle strips obtained from the gastric corpus of guinea pigs developed spontaneous rhythmic contractile activity under the experimental organ-bath conditions. The contractile force generated by the preparations was continuously recorded using an isometric force transducer. For quantitative evaluation of the spontaneous contractile activity, the peaks recorded during each 1 min observation period were averaged, providing the F*avr* parameter. The baseline recordings obtained before administration of betahistine or the microsphere formulations served as the reference for assessment of subsequent time-dependent changes in contractile activity. Representative force–time recordings of spontaneous gastric smooth-muscle activity are shown in Figure 1.

### 3.3. Time-Dependent Effect of Free Betahistine on Spontaneous Contractile Activity

**Figure 2:**
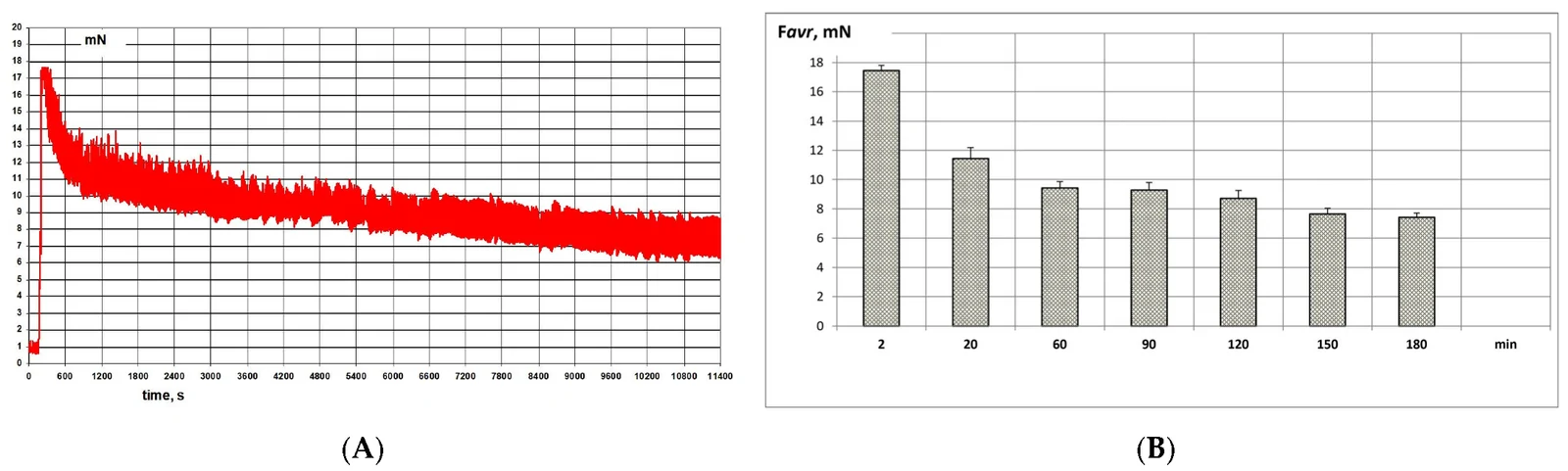
Time-dependent effect of free betahistine on SCA of isolated guinea-pig gastric corpus smooth muscle (**A**), and changes in F*avr* (**B**). F*avr* represents the mean of the contractile peaks recorded during each 1 min observation interval. Data are presented as mean ± SD; *n* = 12 smooth-muscle preparations per treatment group, obtained from six animals. Statistical comparisons were performed at the preparation level without adjustment for within-animal clustering. *p* < 0.05 vs. the response at 2 min (repeated-measures ANOVA followed by Bonferroni’s post hoc multiple-comparison test).

Application of free betahistine at a final concentration corresponding to 10 mg/20 mL (0.5 mg/mL) produced a pronounced early effect on the spontaneous contractile activity of the isolated gastric smooth-muscle preparations. The response was characterized by changes in the magnitude of contractile force and in the temporal pattern of spontaneous contractions. The changes in F*avr* following administration of free betahistine were evaluated relative to the corresponding baseline values obtained from the same preparation (Figure 2). At 2 min after administration, the mean F*avr* was 17.44 ± 0.37 mN. This value decreased to 11.43 ± 0.77 mN at 20 min and to 9.42 ± 0.45 mN at 60 min. At the subsequent observation times, F*avr* remained progressively lower, reaching 9.27 ± 0.52 mN, 8.71 ± 0.55 mN, 7.67 ± 0.38 mN and 7.43 ± 0.30 mN at 90, 120, 150 and 180 min, respectively. F*avr* was significantly lower than the initial 2 min response at all subsequent observation times (*p* < 0.05). Thus, the F*avr* observed at 180 min represented approximately 42.6% of the value recorded at 2 min, corresponding to an overall decrease of approximately 57.4% during the observation period. The free-betahistine response therefore demonstrated a characteristic early maximal response followed by a statistically significant and sustained attenuation of contractile activity.

### 3.4. Time-Dependent Effect of Betahistine-Loaded Microspheres on Spontaneous Contractile Activity

**Figure 3:**
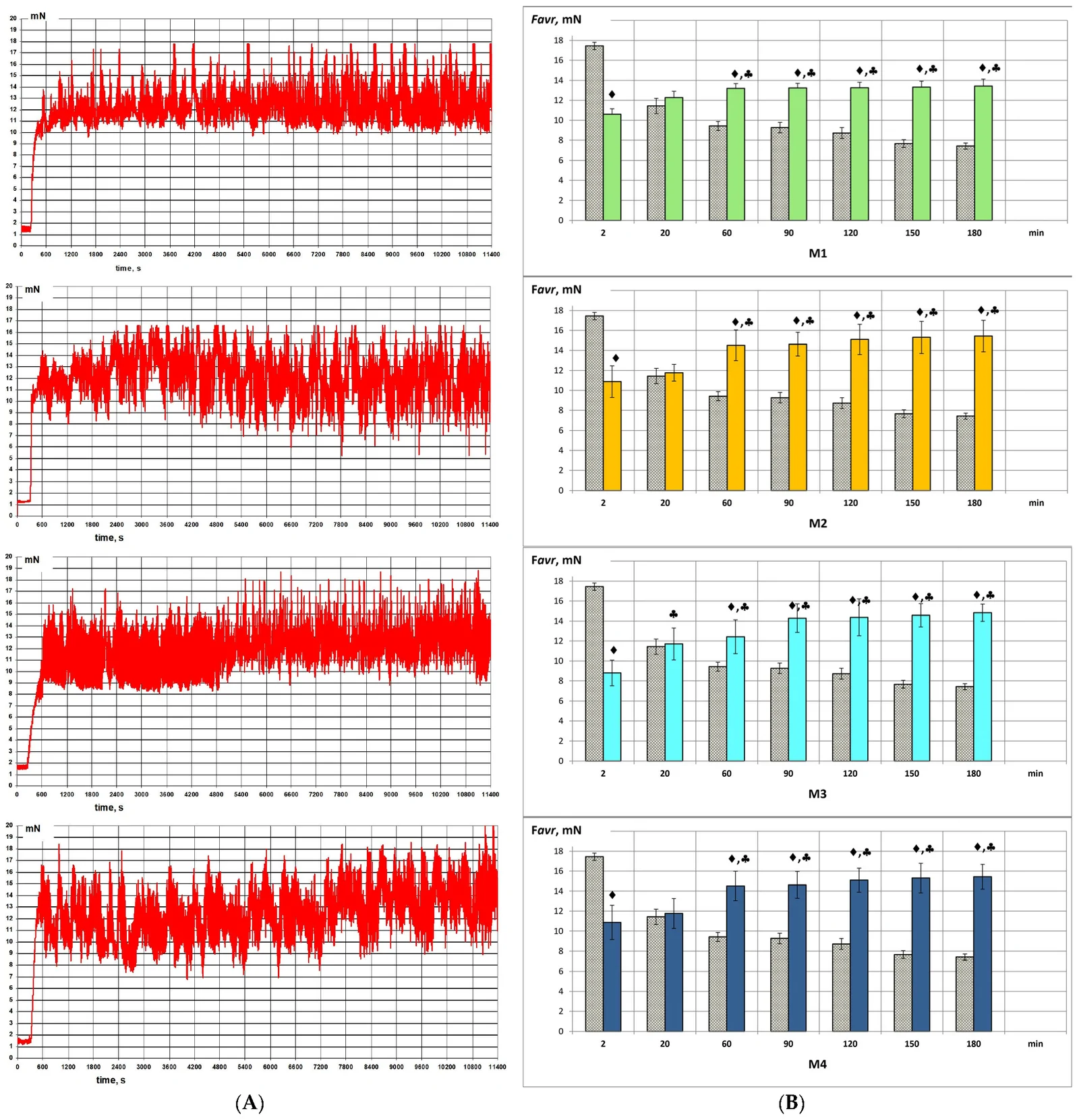
Time-dependent changes in spontaneous contractile activity following administration of betahistine-loaded chitosan microspheres (M1–M4) (**A**) and changes in F*avr* (**B**). F*avr* represents the mean of the contractile peaks recorded during each 1 min observation interval. Data are presented as mean ± SD; *n* = 12 smooth-muscle preparations per treatment group, repeatedly assessed over time, obtained from six animals. Statistical comparisons were performed at the preparation level without adjustment for within-animal clustering. ♦ *p* < 0.05 vs. free betahistine at the corresponding time; ♣ *p* < 0.05 vs. the 2 min response within the same microsphere formulation.

Administration of the betahistine-loaded chitosan microspheres resulted in time-dependent changes in the spontaneous contractile activity of the isolated gastric smooth-muscle preparations. All four microsphere formulations were evaluated using an equivalent nominal betahistine dose of 10 mg. Although the nominal betahistine dose was identical, the mass of microspheres added to the organ bath varied because of differences in drug loading. The temporal profiles of F*avr* demonstrated formulation-dependent changes in the development and persistence of the contractile response. The response profiles of M1–M4 were compared with the response to free betahistine to determine whether incorporation of betahistine into the chitosan matrix altered the temporal characteristics of its pharmacological activity (Figure 3).

In contrast to free betahistine, formulation M1 showed a gradual increase in F*avr* during the observation period. At 2 min, F*avr* was 10.59 ± 0.56 mN and was significantly lower than the corresponding response to free betahistine (*p* < 0.05). F*avr* increased to 12.27 ± 0.63 mN at 20 min, at which point no significant difference from free betahistine or from the initial 2 min M1 response was observed. At 60 min, F*avr* reached 13.19 ± 0.50 mN and remained relatively stable thereafter, with values of 13.24 ± 0.46, 13.26 ± 0.56, 13.33 ± 0.58 and 13.43 ± 0.69 mN at 90, 120, 150 and 180 min, respectively. From 60 to 180 min, F*avr* was significantly higher than both the corresponding free-betahistine response and the initial 2 min M1 response (*p* < 0.05). The overall increase in F*avr* between 2 and 180 min was approximately 26.8%.

Formulation M2 produced a more pronounced increase in F*avr* than M1. F*avr* was 10.87 ± 1.58 mN at 2 min and was significantly lower than the corresponding free-betahistine response (*p* < 0.05). At 20 min, F*avr* increased to 11.76 ± 0.83 mN, with no significant difference from either free betahistine or the initial 2 min M2 response. A marked increase was observed by 60 min, when F*avr* reached 14.51 ± 1.52 mN. At 90, 120, 150 and 180 min, the corresponding values were 14.62 ± 1.19, 15.09 ± 1.51, 15.30 ± 1.60 and 15.43 ± 1.57 mN, respectively. From 60 to 180 min, F*avr* was significantly higher than both the corresponding free-betahistine response and the initial 2 min M2 response (*p* < 0.05). The overall increase from 2 to 180 min was approximately 42.0%, and M2 exhibited the highest F*avr* among the microsphere formulations at the final observation point.

Formulation M3 exhibited the largest relative increase in F*avr* among the four microsphere formulations. At 2 min, F*avr* was 8.80 ± 1.27 mN and was significantly lower than the corresponding free-betahistine response (*p* < 0.05). F*avr* increased to 11.70 ± 1.60 mN at 20 min; this increase was already significant relative to the initial 2 min M3 response (*p* < 0.05), whereas no significant difference from free betahistine was observed at this time point. At 60 min, F*avr* reached 12.43 ± 1.70 mN, followed by 14.28 ± 1.42, 14.38 ± 1.84, 14.57 ± 1.17 and 14.84 ± 0.86 mN at 90, 120, 150 and 180 min, respectively. From 60 to 180 min, F*avr* remained significantly higher than both the corresponding free-betahistine response and the initial 2 min M3 response (*p* < 0.05). The overall increase from 2 to 180 min was approximately 68.6%, representing the largest relative increase among the investigated formulations.

Formulation M4 also showed a gradual increase in F*avr* throughout the observation period. At 2 min, F*avr* was 12.71 ± 1.71 mN and was significantly lower than the corresponding free-betahistine response (*p* < 0.05). F*avr* increased to 13.12 ± 1.50 mN at 20 min, with no significant difference from either free betahistine or the initial 2 min M4 response. At 60 min, F*avr* reached 13.64 ± 1.46 mN and subsequently increased to 14.15 ± 1.34, 14.58 ± 1.20, 14.91 ± 1.48 and 15.21 ± 1.23 mN at 90, 120, 150 and 180 min, respectively. From 60 to 180 min, F*avr* was significantly higher than both the corresponding free-betahistine response and the initial 2 min M4 response (*p* < 0.05). The overall increase from 2 to 180 min was approximately 19.7%.

### 3.5. Comparative Analysis of the Time-Dependent Favr Profiles

Comparison of the complete F*avr* profiles revealed a marked difference between free betahistine and the microsphere formulations. At 2 min, free betahistine produced the highest F*avr* (17.44 ± 0.37 mN), whereas all microsphere formulations showed significantly lower responses (*p* < 0.05), ranging from 8.80 ± 1.27 mN for M3 to 12.71 ± 1.71 mN for M4. At 20 min, the F*avr* values of the microsphere formulations approached those of free betahistine, and no significant differences between the individual microsphere formulations and free betahistine were observed.

From 60 min onward, this relationship was reversed. At 60 min, F*avr* values for M2 (14.51 ± 1.52 mN), M4 (13.64 ± 1.46 mN), M1 (13.19 ± 0.50 mN) and M3 (12.43 ± 1.70 mN) were all significantly higher than the corresponding value for free betahistine (9.42 ± 0.45 mN; *p* < 0.05). This significant difference was maintained at 90, 120, 150 and 180 min for all four microsphere formulations. At 180 min, F*avr* reached 15.43 ± 1.57, 15.21 ± 1.23, 14.84 ± 0.86 and 13.43 ± 0.69 mN for M2, M4, M3 and M1, respectively, compared with 7.43 ± 0.30 mN for free betahistine. Accordingly, the mean F*avr* values at 180 min were approximately 2.08-, 2.05-, 2.00- and 1.81-fold higher for M2, M4, M3 and M1, respectively, than for free betahistine.

**Figure 4:**
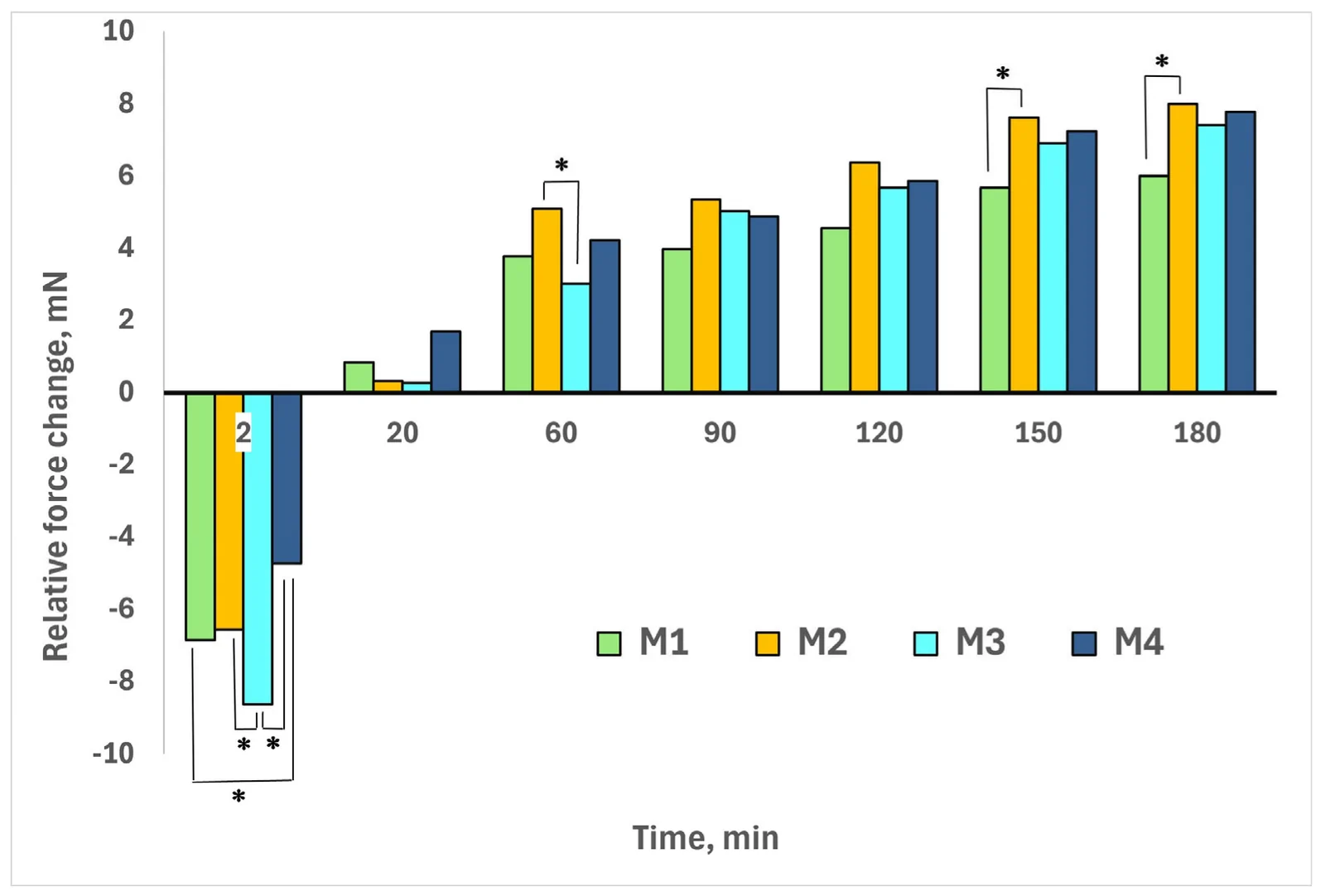
Difference in mean peak contractile force (ΔF*avr*) between each betahistine-loaded chitosan microsphere formulation and free betahistine (BH) at the corresponding observation times. ΔF*avr* was calculated as F*avr*,formulation—F*avr*,BH. Negative values indicate a lower response than free BH, whereas positive values indicate a higher response. Horizontal comparison bars above the columns connect the microsphere formulations being compared; an asterisk (*) above a bar indicates statistically significant pairwise differences between the connected microsphere formulations (*p* < 0.05). Statistical comparisons were performed on the original F*avr* values using one-way ANOVA followed by Bonferroni’s post hoc multiple-comparison test.

Bonferroni post hoc comparisons also revealed significant differences among the microsphere formulations at selected observation times (*p* < 0.05; Figure 4), indicating that the magnitude of the functional response was formulation-dependent at specific stages of the experiment. These differences were not consistent throughout the observation period. No significant pairwise differences were indicated at 20, 90 or 120 min.

### 3.6. Overall Magnitude and Persistence of the Functional Response

The time-dependent F*avr* profiles demonstrated marked differences in the magnitude and persistence of the functional response between free betahistine and the microsphere formulations. Whereas the response to free betahistine progressively declined after its early maximum, all four microsphere formulations showed sustained increases in F*avr*, with values from 60 to 180 min remaining significantly higher than both their respective 2 min responses and the corresponding responses to free betahistine (*p* < 0.05).

The magnitude of the increase between 2 and 180 min differed among the microsphere formulations. M3 exhibited the largest relative increase (68.6%), followed by M2 (42.0%), M1 (26.8%) and M4 (19.7%). In absolute terms, the increase in F*avr* was also greatest for M3 (+6.04 mN), followed by M2 (+4.56 mN), M1 (+2.84 mN) and M4 (+2.50 mN). At 180 min, M2 exhibited the highest F*avr*, followed closely by M4 and M3, whereas M1 showed the lowest value among the microsphere formulations. However, the magnitude of the functional response did not follow the rank order of drug loading: M3, which had the lowest drug-loading value, showed the greatest relative increase in F*avr*, whereas M4, despite having the highest drug loading, did not produce the highest final F*avr*.

### 3.7. Integrated Descriptors of the Time-Dependent Functional Response

The F*avr* time-course profiles were further characterized using the maximum observed F*avr* (F*avr*,max), the time at which F*avr*,max was observed, and the area under the F*avr*–time curve from 2 to 180 min (AUC_2–180_) (Table 2). These parameters provided complementary information on the magnitude, temporal development and persistence of the functional response.

Free betahistine exhibited the highest F*avr*,max (17.44 mN), which occurred at the first observation point, 2 min. The corresponding F*avr*,max values for M1, M2, M3 and M4 were 13.43, 15.43, 14.84 and 15.21 mN, respectively. In all microsphere groups, F*avr* continued to increase up to the final observation point. Therefore, the true maximum response and the corresponding time to maximum could not be established within the 180 min observation period and were considered not to have been reached.

Despite its pronounced early response, free betahistine exhibited the lowest AUC_2–180_ (1698.83 mN·min). All microsphere formulations showed higher integrated responses, with AUC_2–180_ values of 2309.14, 2528.47, 2373.05 and 2509.62 mN·min for M1, M2, M3 and M4, respectively. Among the microsphere formulations, M2 showed the highest AUC_2–180_, closely followed by M4.

### 3.8. Relationship Between Betahistine Release and Functional Response

**Figure 5:**
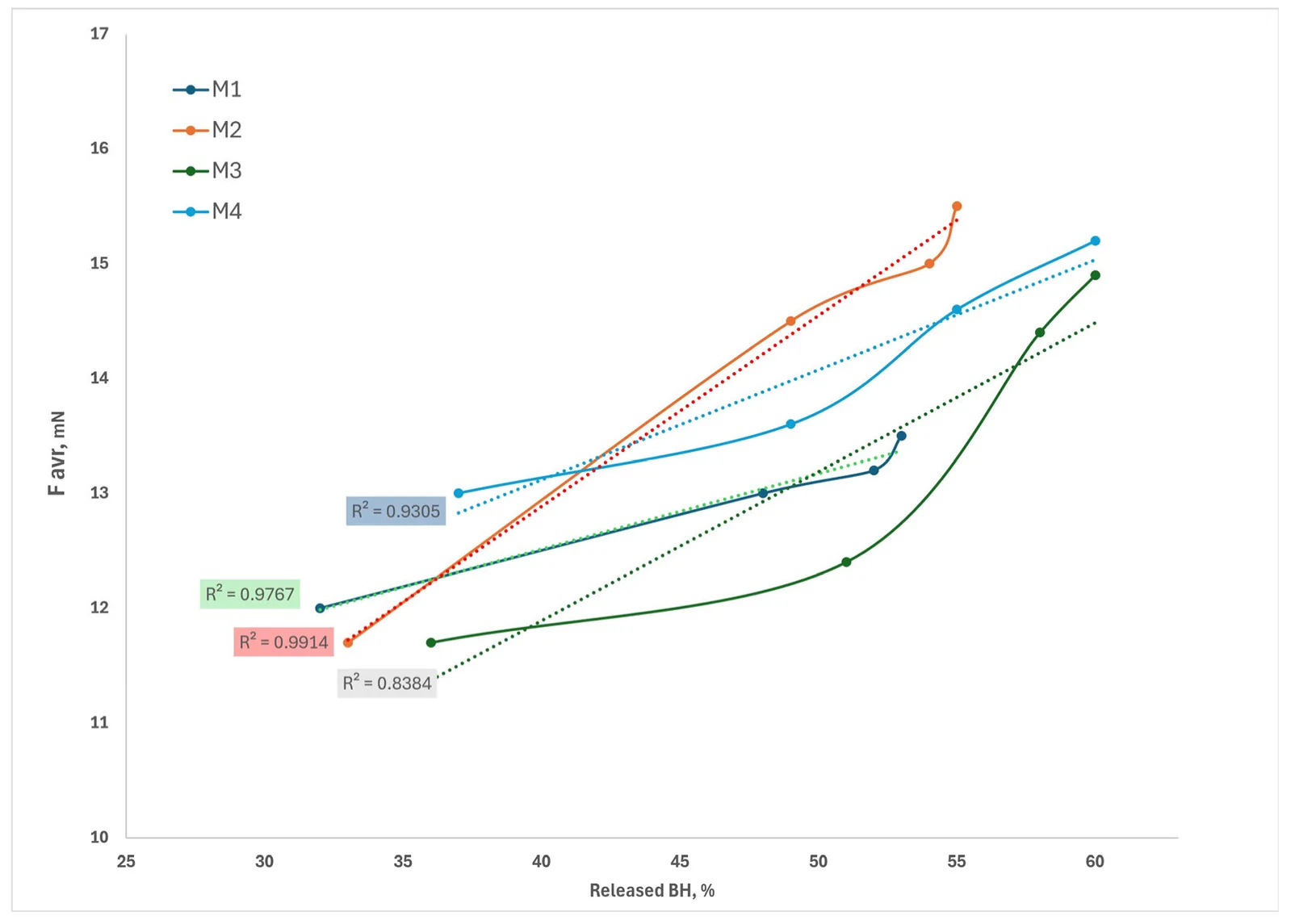
Time-matched relationship between cumulative betahistine (BH) release and functional response of chitosan microsphere formulations M1–M4 (solid lines). F*avr* values obtained in the present ex vivo experiments were paired with the previously determined cumulative BH release values for the corresponding formulation at the four common observation times (20, 60, 120 and 180 min). Dotted lines represent linear regression fits, with the corresponding coefficients of determination (R^2^) shown for each formulation. Previously determined BH-release data are based on [11]. Regression analyses were based on four common observation times per formulation. The reported R^2^ values describe exploratory associations and do not indicate a validated predictive or causal relationship.

To examine the relationship between betahistine release and functional activity, F*avr* values obtained in the present ex vivo experiments were paired with the previously determined cumulative betahistine release values for the corresponding microsphere formulation at the four common observation times (20, 60, 120 and 180 min). The resulting time-matched release–response profiles are presented in Figure 5.

A positive relationship between cumulative betahistine release and F*avr* was observed for all four microsphere formulations. Linear regression analysis of the four time-matched data pairs yielded coefficients of determination (R^2^) of 0.9767, 0.9914, 0.8384 and 0.9305 for M1, M2, M3 and M4, respectively. The highest R^2^ was obtained for M2, followed by M1 and M4, whereas M3 showed a comparatively lower degree of linear fit.

Although positive release–response associations were observed within individual formulations, their interpretation is limited by the small number of time-matched observations (four per formulation) and the independent execution of the in vitro release and ex vivo functional experiments. Furthermore, because both variables changed over time, their apparent association may partly reflect common temporal dependence. Consequently, the regression results should be considered descriptive and exploratory rather than evidence of a validated, predictive or causal in vitro–ex vivo relationship.

## 4. Discussion

The present study provides functional evidence that incorporation of betahistine into chitosan microspheres substantially modifies the temporal profile of its biological activity in isolated guinea-pig gastric smooth muscle. Whereas free betahistine produced a pronounced early response followed by a statistically significant and sustained decline, all four microsphere formulations produced lower initial responses followed by gradual increases in contractile activity. From 60 min onward, F*avr* was significantly higher for all microsphere formulations than for free betahistine at the corresponding observation times. Moreover, time-matched comparison of cumulative betahistine release and F*avr* at 20, 60, 120 and 180 min revealed positive, formulation-dependent release–response associations. Together, these findings provide functional evidence consistent with prolonged availability of pharmacologically active betahistine from the chitosan microsphere formulations.

### 4.1. Distinct Temporal Profiles of Free and Betahistine-Loaded Microsphere Formulations

Free betahistine and the betahistine-loaded microsphere formulations produced clearly distinct temporal patterns of functional activity. Free betahistine elicited its highest response at the earliest observation point, followed by a significant progressive decline throughout the experimental period. In contrast, all four microsphere formulations showed lower initial responses but gradually increasing contractile activity, with F*avr* remaining significantly higher than the corresponding free-betahistine response from 60 to 180 min.

These contrasting profiles are consistent with the different modes of drug presentation to the tissue. Free betahistine was administered in solution and was immediately available in the organ-bath medium. In contrast, betahistine in the microsphere formulations was associated with the chitosan matrix, and its availability to the tissue was expected to depend partly on release from the carrier. This provides a plausible explanation for the rapid but transient response observed with free betahistine and the more gradually developing and sustained responses associated with the microsphere formulations.

The integrated response parameters further support this distinction. Although free betahistine produced the highest F*avr*,max, it exhibited the lowest AUC_2–180_. Conversely, all four microsphere formulations showed lower early responses but higher integrated functional responses over the 180 min observation period. Furthermore, F*avr* continued to increase up to the final observation point in all microsphere groups, indicating that a clear maximum response had not been reached within the experimental period. Thus, incorporation of betahistine into the microsphere formulations was associated with a redistribution of the functional response over time, from a pronounced early effect toward a more sustained pattern of activity.

### 4.2. Relationship Between Previously Determined Release and Functional Response

The previously obtained in vitro release profiles provide an important framework for interpreting the present functional findings. To examine the release–response relationship more directly, F*avr* values were paired with the corresponding cumulative betahistine-release values at the four common observation times of 20, 60, 120 and 180 min. Linear regression analysis demonstrated positive, formulation-dependent associations between cumulative release and functional response, with R^2^ values of 0.9767, 0.9914, 0.8384 and 0.9305 for M1, M2, M3 and M4, respectively. Thus, the functional response generally increased as cumulative betahistine release increased, although the strength and pattern of the linear fit differed among formulations.

These findings strengthen the interpretation that the gradually developing contractile responses were temporally associated with progressive betahistine liberation from the chitosan matrix. However, the release–response relationship was not identical across formulations. M3, for example, showed the highest cumulative release at the later observation times, whereas M2 produced the highest final F*avr* and the greatest AUC_2–180_. This indicates that cumulative release alone does not fully account for the magnitude of the observed biological response.

This distinction is consistent with the different information provided by the two experimental approaches. Cumulative release describes drug liberation from the polymeric matrix into the release medium, whereas F*avr* reflects a downstream biological response that depends on drug availability to the tissue and subsequent pharmacological interaction. The observed differences among formulations may therefore reflect not only release kinetics but also formulation-dependent drug presentation, diffusion and tissue responsiveness.

Importantly, the present analysis should not be interpreted as establishing a formal in vitro–ex vivo correlation. The release and functional experiments were performed separately. Only four common time points were available for regression analysis, and betahistine concentrations in the organ-bath medium were not measured directly. Accordingly, the high R^2^ values support a formulation-specific quantitative association between cumulative release and functional response, but do not by themselves demonstrate a direct causal or predictive relationship.

### 4.3. Biological Activity Associated with Betahistine Release

A principal objective of the present functional study was to determine whether betahistine incorporated into the chitosan microsphere formulations remained pharmacologically active after its release from the carrier matrix. The isolated guinea-pig gastric smooth-muscle preparation provided a responsive biological system in which such activity could be detected as changes in contractile force. All four betahistine-loaded microsphere formulations produced measurable and sustained increases in F*avr*, demonstrating that the formulations were associated with preserved pharmacological activity over the 180 min observation period.

The functional response observed with the microsphere formulations is consistent with the release of biologically active betahistine from the chitosan matrix. This interpretation is supported by the progressive increase in F*avr* over time, the significantly higher responses relative to free betahistine from 60 to 180 min, and the positive time-matched associations between cumulative betahistine release and F*avr*. Importantly, the present assay does not directly quantify the amount of betahistine released into the organ-bath medium; rather, it provides functional evidence that the microsphere formulations can generate a sustained biological effect compatible with continued drug availability.

Such functional assessment complements conventional in vitro release testing because it addresses a different aspect of delivery-system performance. In vitro release studies characterize the kinetics and extent of drug liberation from the carrier, whereas the ex vivo smooth-muscle assay evaluates whether the drug associated with the formulation remains capable of eliciting a pharmacological response in biological tissue. The combined use of these approaches therefore provides a more complete characterization of the microsphere formulations than either release testing or functional assessment alone.

### 4.4. Drug Loading and Functional Performance

Drug loading alone did not determine the magnitude or temporal development of the functional response. M4 exhibited the highest drug loading (54.0%), whereas M3 had the lowest (36.8%). Nevertheless, M3 showed the greatest relative increase in F*avr* between 2 and 180 min, while M2 produced the highest F*avr* at 180 min and the greatest AUC_2–180_. Thus, the rank order of functional performance did not correspond to the rank order of drug loading.

Drug loading describes the proportion of betahistine incorporated into the microsphere formulation. In contrast, the functional response depends on the amount and temporal availability of pharmacologically active drug at the tissue. Accordingly, a formulation with a higher drug-loading value would not necessarily be expected to produce a greater response at a particular observation time, particularly because equivalent nominal amounts of betahistine, rather than equivalent masses of microspheres, were used in the functional experiments.

Formulation characteristics may additionally influence the temporal availability of betahistine from the polymeric matrix. Differences in chitosan content, drug-to-polymer ratio, matrix structure and drug–polymer interactions may contribute to formulation-dependent release behavior and, consequently, to differences in the development of the functional response [16]. In the present study, however, these factors were not evaluated independently and their individual contributions to the observed pharmacological profiles cannot be determined.

### 4.5. Methodological Considerations and Limitations

Establishing quantitative relationships between drug release and subsequent biological performance remains methodologically challenging for microsphere-based delivery systems [17]. Because the release and functional experiments were performed separately and shared only a limited number of observation times, the observed associations cannot be interpreted as a formal in vitro–ex vivo correlation. Therefore, the observed associations should be interpreted as descriptive, formulation-specific release–response relationships rather than as evidence of a validated in vitro–ex vivo correlation.

An additional consideration in interpreting these relationships is the experimental dose-normalization approach. The quantities of each microsphere formulation were adjusted to provide the same nominal amount of betahistine (10 mg), allowing the functional responses of M1–M4 to be compared at an equivalent nominal drug dose. However, differences in drug loading resulted in microsphere masses ranging from 18.52 mg for M4 to 27.17 mg for M3.

This variation in carrier mass represents a further limitation of the experimental design. Unloaded chitosan-microsphere controls were not included in the experimental design. Therefore, potential effects of the polymeric carrier, its composition and the different amounts of microsphere material on the observed contractile responses cannot be excluded. Consequently, differences in functional activity among M1–M4 cannot be attributed exclusively to differences in betahistine release. Accordingly, the sustained contractile responses observed with M1–M4 should be interpreted as the overall functional effects of the drug-loaded formulations rather than as effects attributable exclusively to betahistine release. This distinction is particularly important when comparing individual formulations, because differences in drug loading resulted in different amounts of microsphere material being introduced into the organ bath.

A further limitation is that betahistine concentrations in the organ-bath medium were not measured directly during the functional experiments. Therefore, the amount of betahistine present in the organ bath could not be directly related to the magnitude of the observed contractile response.

An additional statistical limitation concerns the hierarchical structure of the experimental data. Although preparations from all six animals were represented in each treatment group, individual animal identifiers were not retained. Consequently, potential within-animal correlations could not be incorporated into the statistical analysis. This may have affected the effective number of independent observations and the precision of the reported statistical comparisons. The statistical significance of between-group differences should therefore be interpreted with caution.

Finally, the isolated-tissue model does not reproduce the processes of absorption, distribution, metabolism and elimination that determine drug exposure in vivo. The present findings should therefore be interpreted as evidence of formulation-associated pharmacological activity under controlled ex vivo conditions rather than as a direct prediction of in vivo pharmacokinetic or therapeutic performance.

A further limitation is that betahistine concentrations in the organ-bath medium were not measured directly during the functional experiments. Therefore, direct correspondence between the amount of betahistine present in the organ bath and the magnitude of the contractile response could not be established.

### 4.6. Integrated Characteristics of the Functional Response

Analysis of F*avr*,max and AUC_2–180_ provides an integrated view of the marked differences in the temporal characteristics of the responses to free betahistine and the betahistine-loaded microsphere formulations. Free betahistine produced the highest observed F*avr*,max, which occurred at the earliest observation point, but exhibited the lowest AUC_2–180_. In contrast, all four microsphere formulations showed lower observed F*avr*,max values but higher AUC_2–180_ values, reflecting a more sustained functional response over the 180 min observation period.

Importantly, F*avr* continued to increase up to 180 min in all microsphere groups. Therefore, the F*avr*,max values reported for M1–M4 represent the highest values observed within the experimental period rather than confirmed maximal responses. Similarly, 180 min should not be interpreted as the time to maximum response, because a clear response maximum was not reached. A longer observation period would be required to determine the actual maximum and the subsequent decline of the functional response.

Taken together, F*avr*,max and AUC_2–180_ emphasize complementary aspects of the response profile. F*avr*,max reflects the greatest response observed within the experimental interval, whereas AUC_2–180_ reflects the overall magnitude and persistence of the functional response over time. The comparatively high AUC_2–180_ values of the microsphere formulations, despite their lower early responses, therefore, further support the sustained nature of their functional activity.

### 4.7. Biological Interpretation and Relevance of the Organ-Bath Model

The isolated guinea-pig gastric smooth-muscle preparation provided a sensitive functional model for detecting pharmacological activity associated with the betahistine-loaded microsphere formulations. Conventional release testing quantifies drug liberation into a release medium. In contrast, the organ-bath assay measures changes in contractile activity following direct exposure of viable tissue to the formulation. It therefore provides complementary information on whether the drug associated with the delivery system remains capable of eliciting a measurable pharmacological response.

The suitability of this preparation is supported by the established responsiveness of guinea-pig gastric smooth muscle to histaminergic stimulation. Previous studies have demonstrated H1-receptor-mediated contractile responses to histamine in guinea-pig gastric tissue, providing a pharmacological basis for using changes in contractile force as a functional endpoint in the present model [8,9,10].

The model should nevertheless be regarded as a functional bioassay rather than as a simulation of the clinical route of betahistine administration or its systemic pharmacological effects. Its principal value in the present study is the ability to distinguish the rapid, declining response to freely available betahistine from the gradually developing and sustained responses associated with the microsphere formulations under controlled experimental conditions. In this context, the organ-bath approach bridges conventional physicochemical release characterization and biological assessment by providing direct evidence of a tissue-level functional response.

### 4.8. Overall Interpretation

Taken together, the present findings demonstrate distinct temporal patterns of functional activity for free betahistine and the betahistine-loaded chitosan microsphere formulations in the investigated ex vivo model. Free betahistine produced a rapid and pronounced response, with the highest F*avr* observed at the earliest investigated time point, followed by progressive attenuation. In contrast, the microsphere formulations produced lower initial responses followed by gradual increases in contractile activity that persisted throughout the 180 min observation period, without reaching a clear maximum.

The sustained functional profiles were consistent with the previously established prolonged-release characteristics of the corresponding microsphere formulations. Moreover, time-matched analysis at 20, 60, 120 and 180 min revealed positive formulation-dependent associations between cumulative betahistine release and F*avr*. Although these relationships support a temporal link between progressive drug liberation and development of the functional response, they should be interpreted in the context of separate release and functional experiments and the limited number of common observation times.

Overall, the findings provide functional evidence consistent with sustained availability of pharmacologically active betahistine from the chitosan microsphere formulations. The data do not establish a direct causal relationship between microsphere incorporation and the prolonged functional response. Nevertheless, the microsphere formulations exhibited a distinct temporal pattern of pharmacological activity, characterized by a lower initial response and a more sustained effect over time.

From a pharmaceutical technology perspective, the study illustrates the value of complementing conventional in vitro release characterization with a functional biological assay. The two approaches provide distinct but complementary information. Release testing characterizes drug liberation from the carrier, whereas the ex vivo assay evaluates the biological response to the drug-loaded formulation. Their combined application therefore provides a more comprehensive assessment of modified-release microsphere systems than either approach alone.

## 5. Conclusions

The present study demonstrates distinct temporal patterns of functional activity for free betahistine and betahistine-loaded chitosan microsphere formulations in isolated guinea-pig gastric smooth muscle. Free betahistine produced a pronounced early response followed by progressive attenuation, whereas all four microsphere formulations produced lower initial responses followed by gradually increasing and sustained contractile activity throughout the 180 min observation period. The higher AUC_2–180_ values of all microsphere formulations further reflected the persistence of their functional responses compared with free betahistine. Time-matched analysis at 20, 60, 120 and 180 min revealed positive formulation-dependent associations between previously determined cumulative betahistine release and F*avr*. These exploratory findings indicate a temporal relationship, without establishing a causal or predictive validity. Drug loading alone, however, did not determine the magnitude or temporal pattern of functional activity.

Overall, the findings provide functional evidence consistent with sustained availability of pharmacologically active betahistine from the chitosan microsphere formulations and demonstrate the value of complementing conventional in vitro release characterization with an ex vivo functional assay. This combined approach provides a more comprehensive assessment of modified-release microsphere systems by integrating information on drug liberation with evidence of tissue-level pharmacological activity. The absence of unloaded microsphere controls and the inability to account for within-animal correlations should be considered when interpreting these findings. Nevertheless, establishing a definitive causal relationship between betahistine release and the observed pharmacological response requires further investigation.

## Figures and Tables

**Table 1:** Composition and drug-loading characteristics of betahistine-loaded chitosan microspheres and calculated microsphere mass corresponding to a nominal 10 mg betahistine dose. Free betahistine (10 mg) served as the reference treatment; M1–M4 were compared at equivalent nominal drug amounts rather than equivalent microsphere or chitosan masses.

Formulation	BH (mg)	Chitosan (mg)	BH/Chitosan Ratio	Drug Loading (%)	Microsphere Mass Corresponding to 10 mg BH (mg)
M1	100	100	1:1	47.4	21.10
M2	100	150	1:1.5	46.8	21.37
M3	100	200	1:2	36.8	27.17
M4	200	200	1:1	54.0	18.52

**Table 2:** Integrated descriptors of the time-dependent functional response of isolated guinea-pig gastric smooth-muscle preparations following administration of free betahistine and betahistine-loaded chitosan microspheres.

Group	F*avr*,max, mN	Time of F*avr*,max, min	AUC_2–180_, mN·min
BH	17.44	2	1698.83
M1	13.43	Not reached within 180 min	2309.14
M2	15.43	Not reached within 180 min	2528.47
M3	14.84	Not reached within 180 min	2373.05
M4	15.21	Not reached within 180 min	2509.62

## Data Availability

The original contributions presented in this study are included in the article. Further inquiries can be directed to the corresponding author.

## Abbreviations

The following abbreviations are used in this manuscript:

BH	Betahistine
DL	Drug loading
AUC	Area under the curve
ACh	Acetylcholine
SCA	Spontaneous contractile activity
